# Gingerol Enhances Osteogenic/Odontogenic Differentiation of Dental Pulp Stem Cells Impaired by Oxidative Stress via the Wnt/β-Catenin Pathway

**DOI:** 10.3390/jfb17060266

**Published:** 2026-06-01

**Authors:** Abdullah Alqarni, Jagadish Hosmani, Naif Abdulrhman Al Fadhil, Nasser Zafer Abdullah AlHamid, Hassan Ahmed Assiri, Rayan Mohammedfarooq Meer, Bandar Yahya Alshehri

**Affiliations:** 1 Department of Oral Diagnosis, Oral Biology & Periodontology, College of Dentistry, King Khalid University, Abha 61421, Saudi Arabia; aawan@kku.edu.sa (A.A.); halmuawad@kku.edu.sa (H.A.A.); 2 Intern Clinic, College of Dentistry, King Khalid University, Abha 61421, Saudi Arabia; nana99199na@gmail.com (N.A.A.F.); nasser1msder@gmail.com (N.Z.A.A.); 3 Department of Preventive Dental Sciences, College of Dentistry, Taibah University, Madinah 42353, Saudi Arabia; rmeer@taibahu.edu.sa; 4 Department of Pediatric Dentistry and Orthodontic Sciences, College of Dentistry, King Khalid University, Abha 61421, Saudi Arabia; balshehri@kku.edu.sa

**Keywords:** dental pulp stem cells, gingerol, oxidative stress, tissue regeneration, Wnt/β-catenin pathway, stem cells

## Abstract

**Background and Objectives:** Dental pulp stem cells (DPSCs) possess significant regenerative potential; however, oxidative stress impairs their viability and osteogenic differentiation. Gingerol, the principal bioactive component of ginger, exhibits antioxidant and cytoprotective properties. This study evaluated the protective effects of gingerol on DPSCs exposed to H_2_O_2_-induced oxidative stress. **Materials and Methods:** DPSCs isolated from extracted human teeth following Institutional Review Board approval and informed consent were exposed to H_2_O_2_-induced oxidative stress and treated with varying concentrations of gingerol. Cell viability, migration, osteogenic activity, mineralization, intracellular ROS accumulation, and Wnt/β-catenin signaling-related gene expression were evaluated using MTT, scratch wound healing assay, Alizarin Red S staining, ROS staining, ELISA, and real-time PCR. **Results:** Gingerol improved DPSC viability, migration, and mineralization under oxidative stress conditions. Increased ALP and BSP expression indicated enhanced osteogenic activity, while reduced ROS accumulation suggested attenuation of oxidative injury. Gingerol also modulated MMP-2 and MMP-9 expression and normalized oxidative stress-associated alterations in inflammatory and Wnt/β-catenin signaling-related gene expression. **Conclusions:** Gingerol demonstrated protective effects against oxidative stress-induced dysfunction in DPSCs and supported osteogenic differentiation. These findings suggest that gingerol may serve as a supportive bioactive candidate for regenerative dental applications; however, further mechanistic and in vivo studies are required to confirm its therapeutic potential.

## 1. Introduction

Dental pulp tissue contains a population of mesenchymal stem cells known as dental pulp stem cells (DPSCs) [1]. These cells possess multilineage differentiation potential and can give rise to osteoblasts, adipocytes, chondrocytes, and odontoblast-like cells, thereby contributing to reparative dentin formation and maintenance of pulp vitality [2]. Their ability to differentiate into odontoblasts—the cells responsible for dentin formation [3]—has positioned DPSCs as a promising therapeutic alternative for the management of pulpitis. In addition, DPSCs exhibit robust angiogenic capacity through the secretion of pro-angiogenic factors and the formation of capillary-like structures [4]. This characteristic further supports their application in regenerative endodontic procedures, including scaffold-based strategies aimed at replacing infected pulp tissue and restoring structural and functional integrity [5].

The osteogenic potential of DPSCs, together with their accessibility, makes them an attractive model for regenerative investigations. However, similar to other mesenchymal stem cells, their regenerative capacity declines with aging [6]. Although the precise age-related alterations in DPSCs remain incompletely understood, modulation of their microenvironment through extrinsic factors has been shown to partially restore their regenerative function [7]. Cellular aging is closely associated with oxidative damage. An imbalance between reactive oxygen species (ROS) production and antioxidant defense mechanisms leads to oxidative stress, a major driver of cellular senescence. Excessive ROS generation induces apoptosis, necrosis, and functional impairment, thereby compromising tissue repair processes [8].

Oxidative stress is characterized by sustained overproduction of ROS, which disrupts mitochondrial function and accelerates cellular degeneration [9]. In DPSCs, elevated ROS levels impair osteogenic and odontogenic differentiation, exacerbate inflammatory responses, and delay tissue recovery. Increased ROS levels and oxidative stress markers, including myeloperoxidase and 8-isoprostane, have been detected in pulpitis tissues, underscoring the pathological relevance of oxidative stress in pulpal disease [10].

Stem cell-based therapy represents a central pillar of regenerative medicine due to its potential to restore damaged tissues. Recent advances emphasize the integration of bioactive natural compounds to enhance stem cell function and improve tissue repair outcomes [10,11]. By combining cellular therapy with targeted molecular modulation, regenerative medicine aims to restore tissue integrity and functionality through biologically guided repair mechanisms.

Natural compounds with antioxidant and bioactive properties have attracted growing interest for their capacity to modulate stem cell behavior under stress conditions. Gingerol, the principal phenolic constituent of *Zingiber officinale*, exhibits well-documented antioxidant, anti-inflammatory, and cytoprotective effects [12,13,14,15]. It has been shown to attenuate oxidative stress and regulate key intracellular signaling pathways, including Wnt/β-catenin signaling. These properties make gingerol a promising candidate for enhancing the osteogenic and odontogenic potential of DPSCs, particularly under oxidative stress conditions.

The canonical Wnt/β-catenin pathway plays a fundamental role in stem cell renewal, proliferation, and differentiation [16]. In dental tissues, Wnt signaling is critical for odontoblast differentiation and reparative dentin formation [17,18]. Activation of Wnt/β-catenin signaling leads to cytoplasmic accumulation of β-catenin, followed by its nuclear translocation and transcriptional activation of downstream target genes. This pathway is essential during multiple stages of tooth morphogenesis [19,20]. Expression of Wnt-responsive genes, such as Axin2, in developing odontoblasts further supports its involvement in odontoblast maturation [21]. Disruption of β-catenin impairs odontoblast differentiation and root formation, whereas its overexpression enhances dentin production. Despite its established role during tooth development, the involvement of Wnt/β-catenin signaling in the generation of new odontoblast-like cells in postnatal tissues remains incompletely understood [22,23]. Moreover, activation of Wnt-responsive stem cell populations has been demonstrated to drive tissue repair and regeneration across multiple mammalian tissues [24].

Matrix metalloproteinases (MMPs), particularly MMP-2 and MMP-9, are involved in extracellular matrix remodeling, matrix turnover, cell migration, and tissue repair. In the dentin–pulp complex, regulated MMP activity contributes to remodeling of dentin extracellular matrix components and may influence odontoblast differentiation, dentin repair, and regenerative responses. Therefore, assessment of MMP-2 and MMP-9, alongside established osteogenic markers such as ALP and BSP, provides additional insight into the matrix remodeling and reparative aspects of DPSC osteogenic/odontogenic differentiation under oxidative stress conditions [25,26,27,28].

In this study, we investigated whether gingerol can restore the osteogenic and odontogenic differentiation capacity of DPSCs impaired by oxidative stress. We hypothesized that gingerol mitigates oxidative stress-induced dysfunction in DPSCs through modulation of the Wnt/β-catenin signaling pathway. By elucidating this mechanism, the present work provides a mechanistic foundation for the potential therapeutic application of gingerol in dental and bone regenerative strategies.

## 2. Materials and Methods

### 2.1. Cell Culture

The study protocol was reviewed and approved by the Institutional Review Board/Ethics Committee (Approval No.: IRB/KKUCODETH/2024-25/013). Human dental pulp tissues were collected only after obtaining written informed consent from all donors in accordance with institutional ethical guidelines and the principles of the Declaration of Helsinki. Human dental pulp stem cells (DPSCs) were isolated from extracted teeth obtained from 5 healthy donors and pooled prior to experimental analysis to minimize donor-related variability. Freshly extracted premolars and third molars were collected from systemically healthy donors undergoing routine orthodontic or therapeutic extractions after obtaining informed consent. Teeth with fully formed roots, absence of dental caries, restorations, periodontal disease, pulpal pathology, periapical lesions, or structural cracks were included in the study. Teeth with evidence of infection, developmental anomalies, previous endodontic treatment, extensive restorations, root resorption, or any systemic condition that could affect stem cell biology were excluded from the study. The coronal portion of each tooth was sectioned, and pulp tissue was gently scraped using a sterile scalpel. The collected tissue was transferred to sterile Petri dishes and washed with Dulbecco’s Modified Eagle Medium (DMEM) supplemented with 10% fetal bovine serum (FBS), penicillin (100 U/mL), and streptomycin (100 μg/mL). Tissue fragments were minced and seeded into tissue culture flasks containing complete DMEM and incubated at 37 °C in a humidified atmosphere with 5% CO_2_.

The culture medium was replaced twice weekly until cells reached confluence. Cells were detached using 0.25% trypsin–0.02% ethylenediaminetetraacetic acid (EDTA) for 3–5 min and subcultured. First- and second-passage cells were used to establish master and working cell banks, respectively. Cells were cryopreserved in medium containing 20% FBS and 10% dimethyl sulfoxide (DMSO) and stored in liquid nitrogen until further expansion for experiments [17].

### 2.2. The Cell Viability (MTT) Assay

Cell viability was assessed using the 3-(4,5-dimethylthiazol-2-yl)-2,5-diphenyltetrazolium bromide (MTT) assay, which measures mitochondrial succinate dehydrogenase activity in viable cells.

DPSCs were seeded in 96-well plates at a density of 5 × 10^3^ cells/well in DMEM containing 10% FBS and 1× antibiotic solution and incubated at 37 °C with 5% CO_2_. After attachment, cells were treated with hydrogen peroxide (H_2_O_2_, 100 μM) and gingerol (20–120 μM) for 24 h.

Following treatment, the medium was removed, and cells were incubated with MTT solution (0.5 mg/mL in serum-free medium) for 4 h at 37 °C. Formazan crystals were dissolved in dimethyl sulfoxide (DMSO), and absorbance was measured at 570 nm using a microplate reader [28]. Cell viability was calculated as:

Cell viability = [O.D of treated cells/O.D of control cells] × 100.

### 2.3. Morphology Study

Based on MTT results, 40 μM gingerol was selected for subsequent experiments. DPSCs (2 × 10^5^ cells/well) were seeded in six-well plates and treated with gingerol (40 μM) for 24 h. After treatment, cells were washed with phosphate-buffered saline (PBS, pH 7.4), and morphological changes were examined using an inverted phase-contrast microscope.

### 2.4. Alizarin Red S Staining for Calcium Deposition

To assess calcium deposition, Alizarin Red S (ARS) staining was performed. Dental pulp stem cells were cultured in osteogenic differentiation medium for the desired period. The osteogenic differentiation medium was prepared using Dulbecco’s Modified Eagle Medium (DMEM) supplemented with 10% fetal bovine serum (FBS), 1% penicillin–streptomycin, 50 μg/mL ascorbic acid, 10 mM β-glycerophosphate, and 100 nM dexamethasone. Cells were maintained in differentiation medium for 14–21 days, with medium replacement every 2–3 days, then washed twice with phosphate-buffered saline (PBS, pH 7.4) and fixed with 4% paraformaldehyde (PFA) (Sigma-Aldrich, St. Louis, MO, USA) for 15 min at room temperature. After rinsing twice with distilled water, the cells were incubated with freshly prepared 2% (*w*/*v*) ARS solution (pH 4.1–4.3) for 30 min under gentle shaking. Excess dye was removed by three washes with distilled water. Stained calcium deposits were imaged using a bright-field microscope. For quantification, bound ARS was solubilized with 10% cetylpyridinium chloride (CPC), and absorbance was measured at 550 nm using a microplate reader (e.g., BioTek Synergy HT, Winooski, VT, USA). All experiments were conducted in triplicate, and representative images were presented [29].

### 2.5. Scratch Wound Healing Assay

The in vitro wound healing was evaluated using a popular in vitro wound healing model, the same as in our previous study. The procedure involved seeding 2 × 10^5^ human DPSCs per well on six-well culture plates. Using a 200 μL tip, a wound was made on the cell monolayer, cleansed with PBS, and then captured on camera using an inverted microscope. Using the same microscope, the injured area was photographed after a 24 h treatment session with gingerol (40 μM) and control cells that were given serum-free growth media [30].

### 2.6. DCF-DA Staining

The dichloro-fluorescein diacetate (DCFDA) labeling method was used to measure the quantity of intracellular ROS. After being cultivated at a density of 1 × 10^5^ cells/mL in 24-well plates, the cells were exposed to either gingerol or gingerol plus H_2_O_2_ (100 μM) for a duration of 24 h. Additionally, each well received 200 μL of DCFDA (10 μM) and was incubated for 20 min. The cells were examined under a fluorescent microscope (10×), the unbound DCFDA was rinsed with sterile PBS, and the intracellular ROS signal was measured [31].

### 2.7. ALP and Bone Sialoprotein Activity Assay

Human DPSCs were seeded in 6-well plates in triplicate at a density of 2 × 10^5^ cells/well and cultured in defined media after reaching about 80% confluency. Following 24 h in culture, the cells were treated with gingerol (40 μM). The GENLISA™ ELISA kit (Krishgen Biosystems, Cerritos, CA, USA) was used to evaluate alkaline phosphatase (ALP) and bone sialoprotein (BSP) activity in gingerol-treated Dental Pulp Stem Cells (DPSCs) according to the manufacturer’s instructions.

### 2.8. MP-2 and MMP-9 Quantification with ELISA

Human DPSCs were grown in a particular medium to roughly 80% confluence after being seeded in triplicate in 6-well plates at a density of 2 × 10^5^ cells/well. Following a 24 h treatment with gingerol (40 μM), the MMP-2 and MMP-9 activity of gingerol-treated DPSCs was measured using the Human Matrix Metalloproteinase 2 and 9 ELISA Kit (Abbkine, Atlanta, GA, USA) in accordance with the manufacturer’s instructions.

### 2.9. Real-Time PCR

The gene expression of apoptotic and wound healing biomarker genes was examined using real-time PCR. After being treated with 40 μM of gingerol, total RNA was extracted using Sigma’s Trizol Reagent in accordance with a normal procedure (Sigma-Aldrich). PrimeScript was used to produce 2 μg of RNA reverse transcription-based cDNA and a first-strand cDNA synthesis kit (TakaRa, Shiga, Japan). Specific primers were used to enhance the targeted genes (Table 1). The PCR reaction was conducted using the GoTaq^®^ qPCR Master Mix (Promega, Madison, WI, USA), which includes SYBR green dye and all the other PCR components. The real-time PCR was carried out using a Biorad CFX96 PCR apparatus (Bio-Rad Laboratories, Inc., Hercules, CA, USA). The fold change was calculated using Schmittgen and Livak’s 2^−∆∆CT^ method, and the data were interpreted using the comparative CT approach [32].

### 2.10. Statistical Analysis

All experiments were performed in triplicate, and data are presented as mean ± standard deviation (SD). Statistical analyses were performed using SPSS software (version 29.0; IBM Corp., Armonk, NY, USA). Differences among groups were analyzed using one-way analysis of variance (ANOVA) followed by Tukey’s post hoc test. A *p*-value < 0.05 was considered statistically significant.

## 3. Results

### 3.1. Cytotoxicity Assessment of Gingerol in H_2_O_2_-Induced DPSCs

The MTT assay was performed to evaluate the cytotoxic effects of H_2_O_2_-induced oxidative stress on DPSCs and to determine the protective potential of gingerol. Exposure to H_2_O_2_ (20–120 μM) resulted in a dose-dependent reduction in cell viability. A significant decrease in viability was observed at 100 μM H_2_O_2_ (*p* < 0.05), indicating marked oxidative stress-induced cytotoxicity. Morphological examination at this concentration revealed cell shrinkage and detachment, consistent with apoptosis-related changes.

Treatment with gingerol (10–80 μM) significantly attenuated H_2_O_2_-induced cytotoxicity and restored cell viability in a concentration-dependent manner. The most pronounced protective effect was observed at 40 μM, where viability approached control levels (*p* < 0.05 vs. H_2_O_2_-treated group). Microscopic analysis demonstrated preservation of normal cellular morphology in gingerol-treated groups compared with H_2_O_2_ alone.

At lower concentrations (10–20 μM), gingerol slightly increased viability relative to untreated controls, suggesting a potential stimulatory effect. However, concentrations above 80 μM showed reduced protective efficacy, with a mild decline in viability, indicating possible dose-related cytotoxicity.

**Figure 1:**
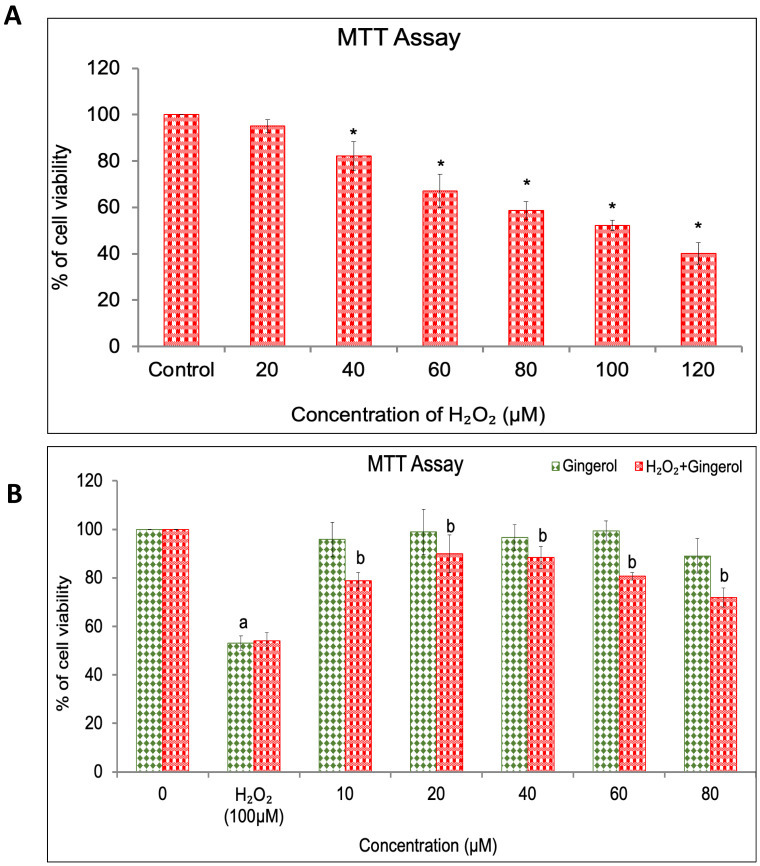
Gingerol’s cytotoxic effects on DPSCs: (**A**) To assess the viability of cells, DPSCs were exposed to H_2_O_2_ and treated with various concentrations of gingerol (20–120 μM) for 24 h. (**B**) Gingerol’s cytotoxicity on H_2_O_2_ was evaluated by treating DPSCs for 24 h with doses ranging from 10 to 80 μM. The MTT assay was used to assess cell viability. Each test was carried out three times, and the findings are shown as Mean ± SD (*n* = 3). * indicates a difference that is statistically significant (*p* < 0.05) when compared to the untreated control group. a—control vs. H_2_O_2_; b—H_2_O_2_ vs. H_2_O_2_ + Gingerol treatment.

Collectively, these findings demonstrate that gingerol confers dose-dependent cytoprotection against oxidative stress in DPSCs, with 40 μM identified as the optimal concentration for subsequent experiments. Representative data and statistical analyses are shown in Figure 1.

### 3.2. Effect of Gingerol on the Morphology of DPSCs Under Oxidative Stress

DPSC morphology was evaluated following exposure to H_2_O_2_ (100 μM) with or without gingerol (40 μM). Cells were seeded in six-well plates (2 × 10^5^ cells/well) and examined after 24 h using an inverted phase-contrast microscope.

Control cells displayed a typical spindle-shaped morphology with elongated cytoplasmic extensions and intact intercellular connections. In contrast, H_2_O_2_-treated cells exhibited marked morphological alterations, including cell shrinkage, rounding, and reduced cell–cell contacts, consistent with oxidative stress-induced damage.

**Figure 2:**
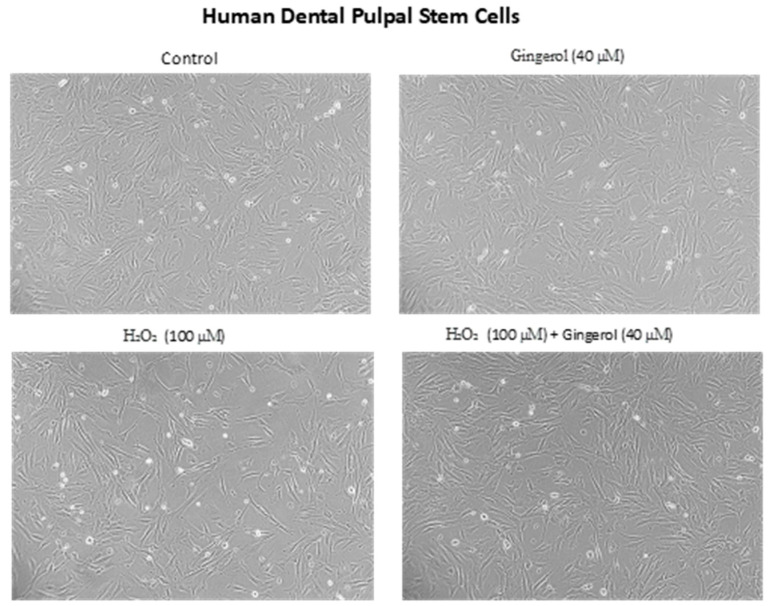
Human DPSCs were treated with gingerol (40 μM) for 24 h, and morphological changes were examined using an inverted phase-contrast microscope. Representative images show cell shape, attachment, and confluency following treatment. Images were captured at 10× magnification.

Co-treatment with gingerol (40 μM) preserved cellular morphology, with cells maintaining elongated extensions and improved structural integrity compared with the H_2_O_2_-treated group. Representative images are shown in Figure 2.

### 3.3. Effect of Gingerol on DPSCs Wound Healing Potential

The effect of gingerol on DPSC migratory behavior under oxidative stress conditions was evaluated using a scratch wound healing assay. Confluent monolayers of DPSCs were mechanically scratched following H_2_O_2_ exposure and subsequently treated with gingerol (40 μM). Untreated cells served as baseline controls, whereas H_2_O_2_-treated cells without gingerol served as the oxidative stress group. Representative images were obtained at 0 and 24 h using an inverted phase-contrast microscope.

**Figure 3:**
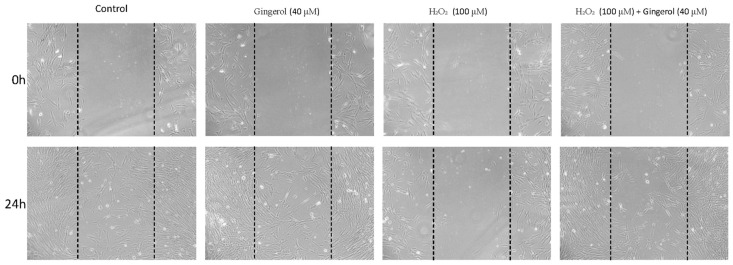
In vitro scratch wound healing assay. H_2_O_2_-induced cells. Human dental pulpal stem cells were injured, and a cell migration assay with and without treatment with gingerol (40 μM) was performed for 24 h. Images were obtained using an inverted phase-contrast microscope (CKX53, Olympus Corporation, Tokyo, Japan). Images were captured at 10× magnification.

After 24 h, H_2_O_2_-treated cells demonstrated delayed wound closure with persistence of a wider scratch area, indicating impaired migratory activity under oxidative stress conditions. In comparison, gingerol-treated cells showed a noticeable reduction in the wound area and improved cellular migration relative to the H_2_O_2_-treated group (Figure 3).

These observations suggest that gingerol partially restores the migratory potential of DPSCs compromised by oxidative stress.

### 3.4. Effect of Gingerol on Intracellular ROS Levels in H_2_O_2_-Induced Oxidative Stress DPSCs

Intracellular reactive oxygen species (ROS) levels were assessed using 2′,7′-dichlorodihydrofluorescein diacetate (DCFDA) staining. Oxidation of DCFDA to fluorescent DCF reflects intracellular ROS accumulation.

Fluorescence microscopy revealed a marked increase in fluorescence intensity in DPSCs treated with H_2_O_2_ (100 μM), indicating elevated ROS levels compared with control cells. H_2_O_2_-treated cells exhibited the highest fluorescence intensity among all groups.

Co-treatment with gingerol (40 μM) significantly reduced fluorescence intensity compared with the H_2_O_2_ group, indicating decreased ROS accumulation. Fluorescence levels in the gingerol co-treated group approached those observed in controls. Cells treated with gingerol alone showed minimal fluorescence, comparable to untreated cells.

**Figure 4:**
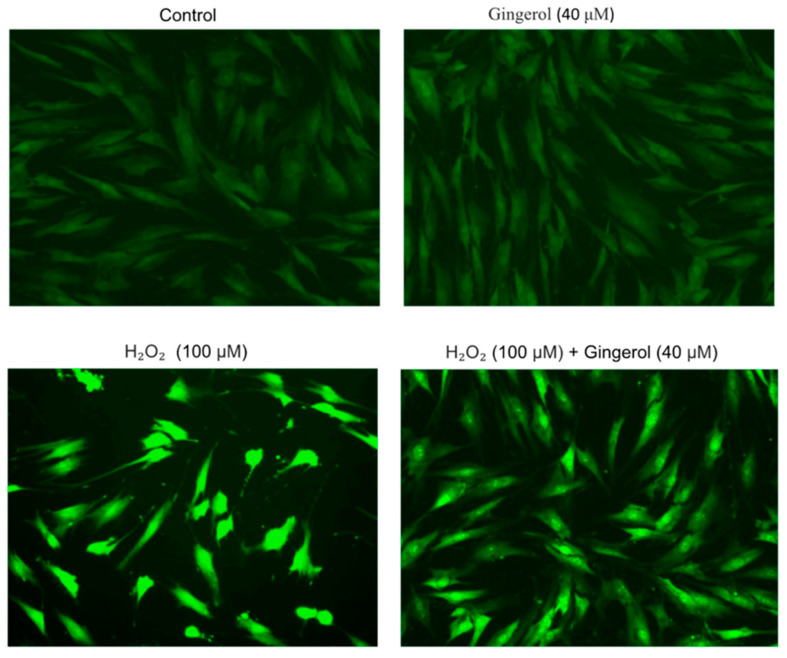
Assessment of ROS level in gingerol-treated H_2_O_2_-induced dental pulpal cells. Cells were treated with indicated concentrations of gingerol or gingerol in combination with H_2_O_2_ (100 µM) for 24 h, and cell images were obtained using an inverted fluorescence microscope. Images were captured at 20× magnification.

These results demonstrate that gingerol attenuates H_2_O_2_-induced ROS generation in DPSCs. Representative fluorescence images and quantitative analysis are shown in Figure 4.

### 3.5. Gingerol Enhances Calcium Deposition in H_2_O_2_-Induced DPSCs

Alizarin Red S (ARS) staining was performed to evaluate calcium deposition during osteogenic differentiation under oxidative stress. DPSCs exposed to H_2_O_2_ exhibited a marked reduction in ARS-stained mineralized nodules compared with controls, indicating impaired extracellular matrix mineralization. The H_2_O_2_-treated group showed faint and sparsely distributed staining.

Co-treatment with gingerol (40 μM) significantly restored mineralized nodule formation compared with the H_2_O_2_ group. Increased staining intensity and broader distribution of ARS-positive deposits were observed in the gingerol-treated cells, indicating enhanced calcium deposition under oxidative stress conditions. Mineralized nodules in this group appeared larger and more densely distributed than in the H_2_O_2_-treated group.

**Figure 5:**
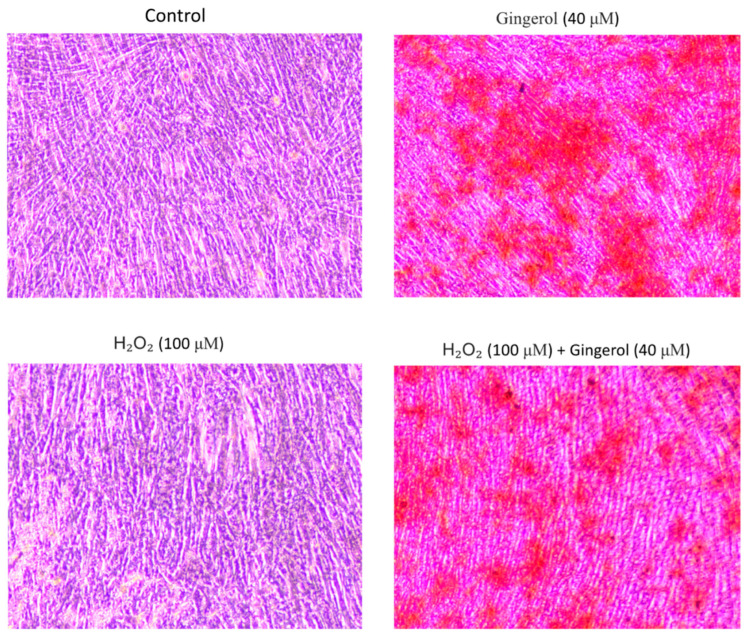
Alizarin Red S staining was used to assess calcium deposition in H_2_O_2_-induced dental pulp stem cells treated with gingerol (40 μM) for 24 h. Images were captured using an inverted phase-contrast microscope. Images were captured at 10× magnification.

Representative images are shown in Figure 5.

### 3.6. Effects of Gingerol on Bone Biomarker and ALP/BSP and MMP2/9 Protein Levels in hDPS Cells

**Figure 6:**
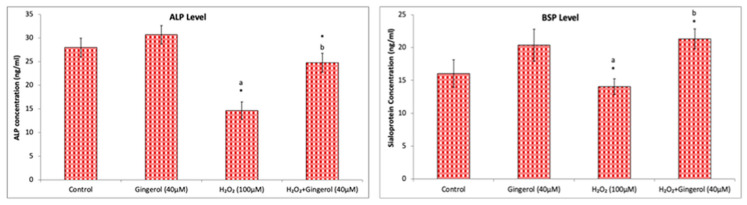
ALP and BSP levels in human dental pulpal stem cells were assessed using an ELISA kit. The conditioned media was collected from control, gingerol-treated, or H_2_O_2_ (100 µM)-treated cells or cells treated with gingerol in combination with H_2_O_2_. Each bar represents the mean ± SD. * Statistically significant difference between two groups (*p* < 0.05); a—Significantly different compared with the control group (*p* < 0.05). b—Significantly different compared with the H₂O₂-treated group (*p* < 0.05).

Gingerol treatment significantly enhanced the expression of osteogenic markers, including alkaline phosphatase (ALP) and bone sialoprotein (BSP), compared with oxidative stress-treated cells (Figure 6). ALP and BSP are widely recognized markers associated with osteogenic differentiation and extracellular matrix mineralization. The observed increase in their expression following gingerol treatment suggests preservation of osteogenic activity in DPSCs under oxidative stress conditions.

**Figure 7:**
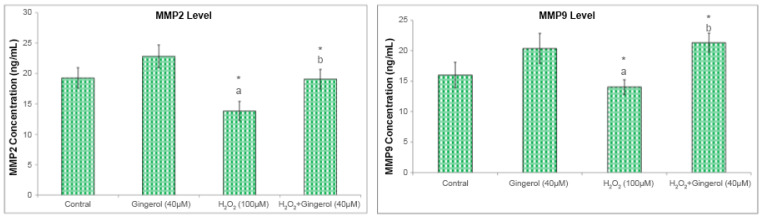
MMP2 and MMP9 levels in human dental pulpal stem cells were assessed using an ELISA kit. The conditioned media was collected from control, gingerol-treated, or H_2_O_2_ (100 µM)-treated cells or from cells treated with gingerol in combination with H_2_O_2_. Each bar represents the mean ± SD. * Statistically significant difference between two groups (*p* < 0.05); a—control vs. other groups; b—H_2_O_2_ vs. H_2_O_2_ + Gingerol treatment.

In addition to osteogenic markers, gingerol treatment also modulated the expression of matrix metalloproteinases MMP-2 and MMP-9 (Figure 7). These enzymes play important roles in extracellular matrix remodeling, cellular migration, and tissue repair processes. Compared with the H_2_O_2_-treated group, gingerol co-treatment resulted in increased expression of both MMP-2 and MMP-9, indicating a potential influence on matrix remodeling dynamics during cellular recovery under oxidative stress conditions.

Taken together, these findings suggest that gingerol supports osteogenic function and modulates extracellular matrix remodeling-associated responses in DPSCs exposed to oxidative stress.

### 3.7. Gingerol Enhances Wound Healing by Regulating Inflammatory Signaling Pathways

**Figure 8:**
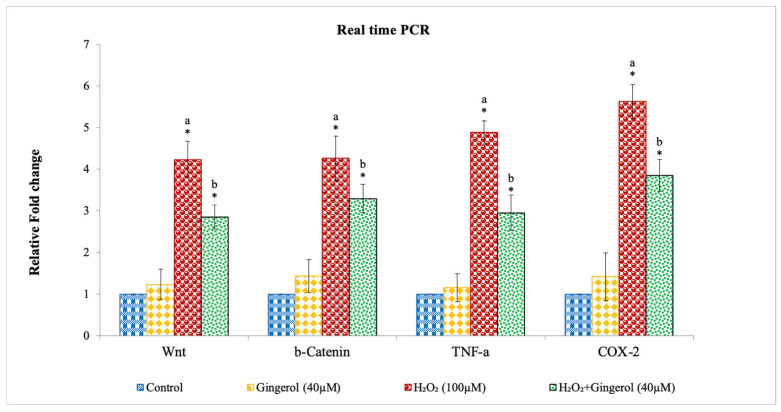
Effect of gingerol or gingerol in combination with H_2_O_2_ (100 µM) on TNF-α, COX-2, Wnt and β-Catenin gene expression in human dental pulpal stem cells. Target gene expression is normalized to GAPDH mRNA expression, and the results are expressed as fold change from the control. Each bar represents the mean + SEM of three independent observations. ‘*’ represents statistical significance between control versus drug treatment groups at *p* < 0.05 level. a—control vs. other groups; b—H_2_O_2_ vs. H_2_O_2_ + Gingerol treatment.

Real-time PCR analysis was performed to evaluate the expression of genes associated with inflammatory responses and Wnt/β-catenin signaling-related pathways under oxidative stress conditions. DPSCs exposed to H_2_O_2_ demonstrated significant upregulation of Wnt/β-catenin signaling-related and inflammatory genes, including β-catenin, TNF-α, and COX-2, compared with the control group (Figure 8).

Treatment with gingerol alone produced only mild modulation of gene expression relative to untreated control cells. Notably, co-treatment with gingerol (40 μM) and H_2_O_2_ significantly attenuated the H_2_O_2_-induced overexpression of β-catenin, TNF-α, and COX-2. Gene expression levels in the co-treated group were markedly reduced compared with the H_2_O_2_-treated group and approached near-baseline levels relative to control cells.

These findings suggest that gingerol modulates oxidative stress-associated transcriptional alterations in inflammatory mediators and Wnt/β-catenin signaling-related genes in DPSCs. However, the present observations are limited to gene expression analysis and do not directly confirm functional activation or suppression of canonical Wnt/β-catenin signaling at the protein level.

## 4. Discussion

The present study explored the effect of gingerol on dental pulp stem cells (DPSCs) exposed to H_2_O_2_-induced oxidative stress, with particular emphasis on cellular viability, migratory behavior, osteogenic differentiation, and stress-associated gene expression changes. The findings suggest that gingerol exerts a protective influence on DPSCs under oxidative stress conditions by reducing intracellular ROS accumulation, improving cellular recovery, and preserving differentiation potential. Rather than indicating a single isolated mechanism, the observed effects appear to reflect a broader modulation of oxidative stress-associated cellular dysfunction and signaling imbalance.

DPSCs are increasingly recognized as an important mesenchymal stem cell population for regenerative dentistry because of their accessibility, proliferative potential, and ability to differentiate into odontogenic and osteogenic lineages [33,34,35,36,37,38]. However, the regenerative performance of these cells is highly sensitive to changes in the local microenvironment. Oxidative stress, particularly when associated with excessive reactive oxygen species (ROS) generation, disrupts cellular homeostasis and compromises survival, migration, and differentiation capacity. This becomes particularly relevant in inflammatory or injured pulpal tissues, where persistent oxidative stress may negatively influence regenerative outcomes.

In the present study, exposure to H_2_O_2_ significantly reduced DPSC viability and produced morphological alterations consistent with oxidative cellular injury. Gingerol treatment improved cell survival in a concentration-dependent manner, with 40 μM demonstrating the most favorable cytoprotective effect. Interestingly, increasing concentrations beyond this range did not proportionally enhance protection, suggesting that the biological effects of gingerol may depend on maintaining an optimal intracellular balance rather than maximal exposure. Similar concentration-dependent antioxidant effects have been reported in other cellular systems treated with gingerol and related phenolic compounds [37,38,39]. The preservation of cellular morphology observed following gingerol treatment further supports its ability to mitigate oxidative stress-associated structural damage.

Oxidative stress also impaired the migratory behavior of DPSCs, as evidenced by delayed wound closure in the scratch assay. This finding is biologically relevant because stem cell migration is essential for tissue repair and regenerative healing. Gingerol-treated cells demonstrated improved wound closure despite oxidative stress exposure, suggesting partial restoration of cellular motility and functional responsiveness. While migration assays cannot fully replicate the complexity of tissue regeneration in vivo, these observations indicate that gingerol may help maintain regenerative competence in stressed cellular environments [40].

One of the central observations of this study was the reduction in intracellular ROS accumulation following gingerol treatment. Elevated ROS levels are known to impair mitochondrial function, alter signaling pathways, and suppress differentiation-related cellular activities. The marked reduction in ROS observed in gingerol-treated groups likely contributed to the preservation of viability and osteogenic activity seen throughout the study. Given the established antioxidant properties of gingerol, the present findings are consistent with its proposed role in stabilizing oxidative microenvironments and limiting ROS-mediated cellular injury.

The influence of oxidative stress on osteogenic differentiation was also evident in the mineralization assays. H_2_O_2_ exposure impaired calcium deposition and reduced extracellular matrix mineralization, whereas gingerol co-treatment restored mineralized nodule formation and enhanced expression of ALP and BSP. These markers are closely associated with osteogenic maturation and matrix mineralization, and their restoration under oxidative stress conditions suggests that gingerol may help preserve differentiation-associated cellular functions despite a hostile oxidative environment [41,42].

In addition to ALP and BSP, the present study evaluated MMP-2 and MMP-9 expression because of their involvement in extracellular matrix remodeling and tissue repair processes. Although matrix metalloproteinases are often associated with matrix degradation and inflammation, controlled MMP activity is also essential for stem cell migration, matrix turnover, and regenerative remodeling within the dentin–pulp complex. The observed modulation of MMP-2 and MMP-9 expression following gingerol treatment may therefore reflect an adaptive remodeling response associated with tissue repair rather than a purely degradative process.

The most conceptually interesting finding of this study relates to the observed changes in Wnt/β-catenin signaling-related gene expression under oxidative stress conditions. Classical models generally associate canonical Wnt/β-catenin signaling with promotion of osteogenesis and differentiation [43,44,45,46]. In contrast, the present findings demonstrated increased expression of Wnt/β-catenin-related genes following H_2_O_2_ exposure despite concurrent impairment of viability and mineralization. At first glance, this appears contradictory to the traditional understanding of Wnt-mediated osteogenic signaling. However, oxidative stress responses in stem cells are often biologically complex and may involve transient or compensatory transcriptional dysregulation rather than effective functional pathway activation.

One possible explanation is that the increased transcriptional expression observed after H_2_O_2_ exposure represents a stress-associated compensatory response rather than productive canonical Wnt signaling. Under oxidative stress conditions, cells may transiently alter signaling-associated gene expression in an attempt to maintain homeostasis or counteract injury. Importantly, increased mRNA expression alone does not necessarily indicate functional stabilization or nuclear translocation of β-catenin, which remains a defining feature of canonical Wnt pathway activation. In this context, the normalization of stress-associated gene expression following gingerol treatment may reflect restoration of cellular signaling balance rather than direct activation or suppression of canonical Wnt signaling itself.

The observed modulation of TNF-α and COX-2 expression further supports the possibility that oxidative stress-associated inflammatory signaling contributes to DPSC dysfunction under H_2_O_2_ exposure. Persistent inflammatory signaling can impair osteogenic differentiation and tissue repair, particularly when coupled with excessive ROS accumulation. Gingerol treatment attenuated the stress-associated overexpression of these inflammatory mediators, which is consistent with the known anti-inflammatory properties of ginger-derived bioactive compounds. Nevertheless, inflammatory markers in the present study should be interpreted as supportive indicators of oxidative stress-associated cellular responses rather than the principal mechanistic focus of the investigation.

Taken together, the findings suggest that gingerol helps preserve DPSC functional integrity under oxidative stress conditions through a combination of antioxidant, cytoprotective, and signaling-modulatory effects. The ability to maintain viability, migration, matrix remodeling activity, and osteogenic differentiation under oxidative challenge may have relevance for regenerative endodontics, pulp tissue engineering, and bone repair strategies where oxidative microenvironments frequently compromise cellular performance.

At the same time, several limitations should be acknowledged. The present mechanistic interpretation is based primarily on transcriptional analysis obtained through real-time PCR. Although changes in Wnt/β-catenin-related gene expression were observed, protein-level validation of β-catenin stabilization and nuclear localization was not performed. Consequently, the current findings should be interpreted as evidence of stress-associated transcriptional modulation rather than definitive confirmation of canonical Wnt pathway activation. Future studies incorporating Western blotting, immunofluorescence analysis, and pathway inhibition approaches would provide greater mechanistic clarity. In addition, immunophenotypic characterization of isolated DPSCs using standard mesenchymal stem cell markers was not performed in the present study and should be incorporated in future investigations. Further in vivo studies will also be necessary to determine the translational relevance, long-term safety, and therapeutic applicability of gingerol-based regenerative approaches.

## 5. Conclusions

Within the limitations of this in vitro study, gingerol demonstrated a protective effect on dental pulp stem cells (DPSCs) exposed to H_2_O_2_-induced oxidative stress. Gingerol treatment reduced intracellular ROS accumulation, improved cellular viability and migratory capacity, and supported osteogenic differentiation, as evidenced by enhanced mineralization and restoration of osteogenic marker expression. In addition, gingerol modulated oxidative stress-associated alterations in inflammatory and Wnt/β-catenin signaling-related gene expression.

The findings suggest that gingerol may help preserve DPSC functional integrity under oxidative stress conditions and could serve as a supportive bioactive candidate for regenerative dental applications. However, the mechanistic observations in the present study are based primarily on transcriptional analysis and should be interpreted cautiously. Further investigations involving protein-level pathway validation, detailed molecular analyses, and in vivo studies are necessary to better clarify the precise biological mechanisms and translational potential of gingerol in regenerative dentistry and tissue engineering.

## Figures and Tables

**Table 1:** Primer sequences were utilized in this study to perform real-time polymerase chain reaction (RT-PCR).

Gene	Forward Primer	Reverse Primer	Tm°C
*Wnt*	5′AGC TGG GTT TCT GCT ACG TT 3′	5′AAT CTG TCA GCA GGT TCG TG 3′	58
*Β-catenin*	5′CGG TCA GGT CAT CAC TAT CG 3′	5′ TTC CAT ACC CAG GAA GGA AG 3′	52
*TNF-α*	5′GCC CAG GCA GTC AGA TCA TCT 3′	5′TTG AGG GGT TGC TAC AAC ATG 3′	60
*COX-2*	5′AAT CGC TGT ACA AGA AGT GG 3′	5′GCA GCC ATT TCT TTC TCT CC 3′	59
*GAPDH*	5′CGACCACTTTGTCAAGCTCA 3′	5′CCCCTCTTCAAGGGGTCTAC 3′	58

## Data Availability

The original contributions presented in the study are included in the article, further inquiries can be directed to the corresponding author.

## Abbreviations

The following abbreviations are used in this manuscript:

ALP	Alkaline phosphatase
AMPK	AMP-activated protein kinase
ARS	Alizarin Red S
BSP	Bone sialoprotein
COX-2	Cyclooxygenase-2
DCFDA	2′,7′-Dichlorodihydrofluorescein diacetate
DPSCs	Dental pulp stem cells
ECM	Extracellular matrix
EMT	Epithelial–mesenchymal transition
GSK3β	Glycogen synthase kinase 3 beta
H_2_O_2_	Hydrogen peroxide
MMP2	Matrix metalloproteinase-2
MMP9	Matrix metalloproteinase-9
MTT	3-(4,5-Dimethylthiazol-2-yl)-2,5-diphenyltetrazolium bromide
NF-κB	Nuclear factor kappa B
Nrf2	Nuclear factor erythroid 2-related factor 2
NPMSCs	Nucleus pulposus-derived mesenchymal stem cells
OS	Oxidative stress
ROS	Reactive oxygen species
TNF-α	Tumor necrosis factor alpha
Wnt	Wingless/Int-1
